# Protective effect of *Loranthus tanakae* Franch. & Sav. on allergic asthma induced by ovalbumin

**DOI:** 10.17221/77/2025-VETMED

**Published:** 2026-05-27

**Authors:** Sin-Hyang Park, So-Won Pak, Woong-Il Kim, Ba-Reun Jin, Young-Kwon Cho, Tae-Won Kim, Je-Won Ko, Joong-Sun Kim, Jong-Choon Kim, A Yeong Lee, In-Sik Shin

**Affiliations:** ^1^College of Veterinary Medicine and BK21 FOUR Program, Chonnam National University, Buk-gu, Gwangju, Republic of Korea; ^2^Center for Convergence Toxicology Research, Korea Institute of Toxicology, Yuseong-gu, Daejeon, Republic of Korea; ^3^College of Health Sciences, Cheongju University, Sangdang-gu, Cheongju-si, Chungbuk, Republic of Korea; ^4^College of Veterinary Medicine and BK21 FOUR Program, Chungnam National University, Daejeon, Republic of Korea; ^5^KM Data Division, Korea Institute of Oriental Medicine, Daejeon, Republic of Korea

**Keywords:** airway inflammation, herbal medicine, immune disease, matrix metalloproteinase-9

## Abstract

Allergic asthma is a widespread disease with elevated eosinophil levels. Although corticosteroids are widely prescribed for allergic asthma, numerous patients experience limited sensitivity and side effects. *Loranthus tanakae* Franch. & Sav., a traditional herbal plant, has anti-inflammatory and antioxidant properties in pulmonary inflammation caused by Asian sand dust and cigarette smoke condensate. To assess the protective effects of *L.* *tanakae*, we examined the influence of an *L.* *tanakae* ethanol extract (LTE) in ovalbumin (OVA)-induced asthma. Mice received intraperitoneal sensitisation with OVA, and challenged using OVA inhalation. LTE was consecutively orally gavaged for 6 days. Following sacrifice, the bronchoalveolar lavage fluid (BALF) and lung tissue was analysed. The LTE treatment considerably reduced inflammatory cell counts, proinflammatory cytokine levels in the BALF, and immunoglobulin E, compared with the OVA group, along with a reduction in airway hyperresponsiveness. The LTE also improved airway inflammation and suppressed mucus hypersecretion in the lung tissues. Additionally, the expression of MMP-9 and activation of ERK, JNK, and p38 were notably diminished in the LTE groups. This study revealed reduced airway inflammation in OVA-induced asthma via suppressing the MMP-9 and mitogen-activated protein kinase-associated factors. Consequently, our findings demonstrated that LTE is suggested as a potential remedy for allergic asthma.

Allergic asthma, recognised as a critical health condition, impacts about 300 million individuals globally, leading to more than 400 000 deaths annually (Yang et al. 2024). It is primarily triggered by exposure to allergens, air pollutants, and chemical agents, and is described by hallmark features including airway hyperresponsiveness (AHR), airway remodelling, and mucus overproduction all of which contribute to clinical symptoms like coughing, sputum production, chest pain, and dyspnoea (Papi et al. 2018). Allergic asthma has a complex twisted pathophysiology that includes several interconnected signalling pathways. Among these, the elevation of Type 2 T-helper (Th2) immune responses is considered a crucial contributor to disease development (Pak et al. 2022). The enhanced Th2 activity promotes the amassment of inflammatory cells, containing macrophages, neutrophils, and eosinophils, in the lung tissues and leads to the amplification of interleukin (IL)-4, IL-5, and IL-13, thereby intensifying allergic inflammatory responses (Lee et al. 2024a). The current therapeutic strategies for allergic asthma include corticosteroids, bronchodilators, and leukotriene receptor antagonists. However, their long-term use is limited by adverse effects such as immunosuppression, nausea, vomiting, and the development of resistance to the drug (Shin et al. 2014; Lowe et al. 2015).

Moreover, bronchodilators do not target the underlying pathogenic mechanisms of allergic asthma, but merely provide symptomatic relief by reducing bronchoconstriction-induced AHR, rendering them insufficient as standalone therapies (Shin et al. 2017). In turn, this has sparked a growing demand for advanced therapeutic strategies that can effectively target the fundamental mechanisms of allergic asthma.

The mitogen-activated protein kinase (MAPK) pathway serves as an integral regulator of immune cell metabolism, inflammatory response modulation, and other physiological processes. Activated MAPKs modulate the inflammatory response by phosphorylating and activating several downstream targets, regulating immune cell functions, and influencing transcription factors. In the context of asthma pathology, the MAPK pathway facilitates airway inflammation by regulating inflammatory cell activation and cytokine secretion (Jiang et al. 2023). Notably, it critically contributes to driving airway remodelling, which is recognised as a core pathological feature of asthma. Airway remodelling is closely linked to heightened matrix metalloproteinase (MMP)-9 expression, a key protease involved in the extracellular matrix degradation (Zou et al. 2019). MMP-9 facilitates airway remodelling through airway inflammation, mucus production, and the destruction of typical alveolar structures in asthma patients. Thus, both the MAPK pathway and MMP-9 are integral to the pathological processes of asthma, contributing to the production of cytokines and chemokines that promote Th2 differentiation and inflammatory responses (Lee et al. 2024a). Therefore, targeting MAPKs and MMP-9 may offer a novel approach to asthma treatment.

*Loranthus tanakae* Franch. & Sav. is a semi-parasitic herbal plant of the Loranthaceae family in some East Asian countries, including South Korea, Japan, and China. *L. tanakae* has traditionally been used to improve the function of the liver, kidneys, and muscles and additionally applied to treat hyperlipidaemia, diabetes mellitus, and cancer (Joo et al. 2019; Park et al. 2022). Previous research has demonstrated that *L. tanakae* exhibits multiple pharmacological properties in respiratory disorders such as chronic obstructive pulmonary disease (COPD) induced by cigarette smoke condensate (CSC) and pulmonary inflammation caused by Asian sand dust (ASD) exposure (Park et al. 2022; Lee et al. 2024b).

Despite its broad pharmacological profile and potential relevance to allergic asthma, no studies to date have specifically examined its therapeutic effects in this context. Furthermore, *L. tanakae* comprises various bioactive substances, including its major constituents, quercitrin and afzelin. Quercetin and kaempferol, the aglycones of quercitrin and afzelin, have been proven to be relatively safe through extensive toxicity studies and long-term *in vivo* experiments (Harwood et al. 2007; Yangzom et al. 2022). These findings suggest that *L.* *tanakae* is likely to be low-toxic even with long-term consumption, although systematic research on the extract’s acute and chronic toxicity remains limited.

Hence, we studied the ingredients of *L.* *tanakae* through high-performance liquid chromatography (HPLC) and scrutinised the therapeutic efficacy of *L.* *tanakae* in allergic asthma through an ovalbumin (OVA)-induced allergic asthma model, aiming to provide preliminary data supporting its clinical potential.

## MATERIAL AND METHODS

### Plant material

The aerial portions of *L.* *tanakae* were sourced through a local herbalist in Repubic of Korea. The plant’s identity was confirmed via genetic analysis by Byeong Cheol Moon, who worked at the Korea Institute of Oriental Medicine (KIOM), and a corresponding voucher specimen (No. 2-16-0335) was preserved in the Korean Herbarium of Standard Herbal Resources. The plant material was air-dried, ground with a blender (HMF-3000S; Hanil Electric, Seoul, Republic of Korea), and sieved to a particle size of 600 μm. Extraction was performed by refluxing the powdered plant material twice for 2 h at 80 °C using 6 l of 70% ethanol. Using chromatography paper (46 × 57 cm), the extract was filtered, and the solvent was evaporated under reduced pressure to ensure complete removal of the ethanol. The extraction process yielded 126.42 g of *L. tanakae* ethanol extract (LET), corresponding to 12.25% (w/w), which was kept at –20 °C. This plant material was previously used in another study (Lee et al. 2024a).

### Animals and ethical declaration

Specific pathogen-free female Balb/c mice (6 weeks old) were obtained from Samtako Co. (Gyeonggi-do, Republic of Korea). The animals were housed under controlled conditions of 22 ± 2 °C, 55 ± 15% relative humidity, and a 12 h light/12 h dark cycle throughout the experimental period. All the animal procedures were reviewed and approved by the Institutional Animal Care and Use Committee of Chonnam National University (CNU IACUC-YB-2021-63) and conducted in accordance with the National Institutes of Health Guidelines for the Care and Use of Laboratory Animals.

### OVA-induced allergic asthma model

**Figure 1:**
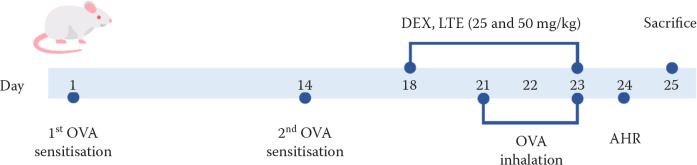
Illustration of the research To induce sensitisation, the animals received OVA injections on days 1 and 14 and were exposed to an OVA solution through aerosol during days 21 to 23. The animals were treated with DEX and LTE via oral gavage on a daily basis from days 18 to 23. The AHR was evaluated on day 24. The sacrifice of the animals was performed on day 25 AHR = airway hyperresponsiveness; DEX = dexamethasone; LTE = *L.* *tanakae* ethanol extract; OVA = ovalbumin

An allergic asthma model was induced as described previously (Lee et al. 2024b) (Figure 1). Mice were sensitised with intraperitoneal OVA (20 μg; Sigma-Aldrich, St. Louis, MO, USA) and aluminium hydroxide (20 mg in 200 μl of PBS, Sigma-Aldrich) on days 1 and 14, followed by aerosolised OVA [1% v/v in phosphate buffered saline (PBS)] exposure via an ultrasonic nebuliser (NE-U12; Omron, Tokyo, Japan) twice daily for 20 min on days 21–23. The airway hyperresponsiveness (AHR) was measured using the FlexiVent system (SCIREQ; Montreal, QC, Canada) after a tracheostomy and exposure to the aerosolised PBS or methacholine (10, 20 and 40 mg/ml, Sigma-Aldrich).

Mice were randomised into five groups (*n *= 6): normal control (NC; PBS for intraperitoneal injection, inhalation, and oral administration), allergic asthma group (OVA; OVA sensitisation and challenge with PBS oral administration), dexamethasone-treated group (DEX; OVA-induced asthma model treated orally with dexamethasone (1 mg/kg in PBS, Sigma-Aldrich)), and two LTE-treated groups (LTE25 and LTE50; OVA-induced asthma model treated orally with 25 or 50 mg/kg of the *L. tanakae* ethanol extract in PBS, respectively). A daily oral gavage of LTE and dexamethasone was performed from days 18 to 23. The selected LTE doses were based on prior experimental data (Lee et al. 2024a).

### Analysis of bronchoalveolar lavage fluids

On day 25, the mice were anaesthetised with intraperitoneal alfaxalone (85 mg/kg; Jurox Pty Ltd., Rutherford, NSW, Australia) and xylazine (10 mg/kg; Bayer Korea, Seoul, Republic of Korea). After the tracheostomy and intubation, the bronchoalveolar lavage fluid (BALF) was collected by instilling 0.7 ml ice-cold PBS twice (total 1.4 ml). The samples were centrifuged (300 × *g*, 10 min, 4 °C), and the supernatants were used to quantify IL-4, IL-5, and IL-13 using Enzyme-Linked Immunosorbent Assay (ELISA) kits (R&D Systems, Minneapolis, MN). Cell pellets were resuspended in 500 μl PBS, and total counts were obtained using an automated cell counter (Cell Countess III; Thermo Fisher Scientific, San Diego, CA, USA). For the differential cell counting, 200 μl was cytocentrifuged (Cytospin; Hanil Science Industrial Co, Ltd., Seoul, Republic of Korea), stained with the Diff-Quik reagent (BIOZOA, Seoul, Republic of Korea), and examined under a light microscope (Leica, Wetzlar, Germany). The proportions of inflammatory cell types were calculated from the total cell counts.

### Analysis of serum

Blood was collected from the caudal vena cava and centrifuged (200 × *g*, 20 min) to obtain the serum. The OVA-specific IgE was quantified by ELISA (BioLegend, San Diego, CA, USA). Briefly, 96-well plates were coated overnight with OVA (10 μg/ml in PBS-Tween-20), blocked, and incubated with serum samples for 2 hours. After washing, Horseradish Peroxidase- (HRP)-conjugated goat anti-mouse IgE antibodies were added, followed by the *o*-phenylenediamine dihydrochloride substrate (Sigma-Aldrich). The plates were incubated in the dark for 10 min, and the absorbance was measured at 450 nm using a spectrophotometer (Bio-Rad, Hercules, CA, USA).

### Histopathological analysis of the lung tissue

Following the BALF collection, the left lung was fixed in 4% paraformaldehyde, embedded in paraffin, sectioned (4 μm), and stained with haematoxylin and eosin (H&E; Sigma-Aldrich) and periodic acid–Schiff (PAS; Sigma-Aldrich) to evaluate the inflammation and mucus production, respectively. For immunohistochemistry (IHC), sections were processed using a commercial kit (Vector Laboratories, Burlingame, CA, USA) with an anti-MMP-9 antibody (1 : 200, ab38898; Abcam, Cambridge, UK) according to the manufacturer’s instructions. Images were captured using a digital slide scanner (Motic, Hong Kong, P.R. China), and quantitative analyses of the inflammation, mucus production, and MMP-9 expression were performed with an image analysis system (IMT i-Solution Inc., Vancouver, BC, Canada).

### Western blotting

Proteins were extracted from the right lung lobes homogenised in a tissue lysis buffer (1/10 w/v; Sigma-Aldrich) containing a protease inhibitor cocktail (Sigma-Aldrich) using a mechanical homogeniser. Protein concentrations were determined using the Bradford assay (Bio-Rad). Western blotting was performed as described previously (Pak et al. 2022). The primary antibodies (1 : 1 000) included *p*-ERK (#9101), ERK (#4695), *p*-JNK (#9251), JNK (B7128), *p*-p38 (#4631), p38 (#9212), and β-actin (#4967) (all from Cell Signaling Technology, Danvers, MA, USA), and MMP-9 (ab38898; Abcam, Cambridge, UK). The band intensities were quantified using a ChemiDoc imaging system (Bio-Rad).

### Statistical analysis

The data were assessed for normality using the Shapiro–Wilk test. Statistical analyses were performed using one-way analysis of variance (ANOVA) followed by Dunnett’s multiple comparison test (GraphPad Prism v8.0; GraphPad Software, San Diego, CA, USA). Data are expressed as the mean ± standard deviation (SD), and the differences were considered significant at *P *< 0.05 or *P *< 0.01.

## RESULTS

### Analysis of ingredient in LTE

**Figure 2:**
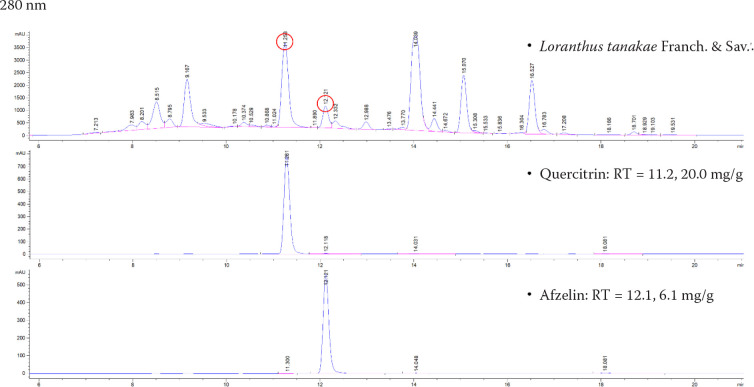
Composition analysis of LTE LTE was analysed using HPLC with detection at 280 nm. The active components are described in the main text HPLC = high-performance liquid chromatography; LTE = *L.* *tanakae* ethanol extract

Marker compounds in LTE were analysed using HPLC-UV (Figure 2). The identity and content of the compounds were validated by comparing their retention times and peak consistency with those of the standard references. Quercitrin and afzelin were identified at the retention times of approximately 11.2 min and 12.1 min, respectively, with the concentrations in LTE determined to be approximately 20.0 mg/g (2.0%) and 6.1 mg/g (0.61%) at a detection wavelength of 280 nm, respectively..

### Effect of LTE on the AHR in asthmatic mice

**Figure 3:**
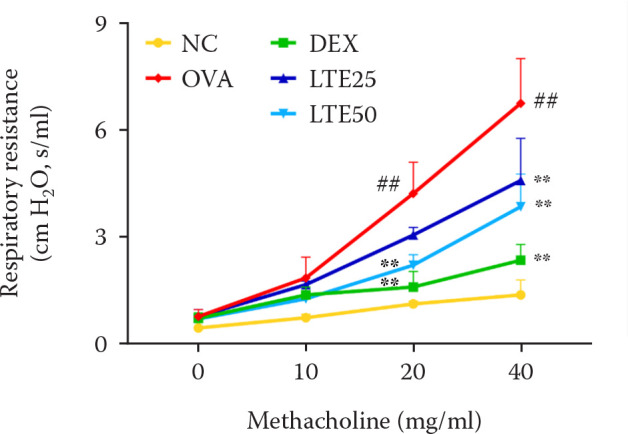
The effect of LTE on the AHR of OVA-inhaled animals The administration of LTE reduced the increased airway hyperresponsiveness in the OVA exposed animals. NC, PBS treatment only; OVA, OVA exposure animals with oral administration of PBS; DEX, OVA exposure animals with oral administration of dexamethasone; LTE25 and LTE50, OVA exposure animals with oral administration (25 and 50 mg/kg, respectively) of LTE The values are displayed as the mean ± SD; *^##^P* *<* 0.01 versus NC, and **P *<* 0.01 versus OVA AHR = airway hyperresponsiveness; DEX = dexamethasone; LTE = *L.* *tanakae* ethanol extract; NC = normal control; OVA = ovalbumin; PBS = phosphate-buffered saline; SD = standard deviation

Airway hyperresponsiveness (AHR), a major indicator of asthma, was significantly higher in the OVA group than in the NC group (Figure 3). The DEX group demonstrated a pronounced reduction in AHR compared with the OVA group. Similarly, the LTE treatment suppressed AHR in a dose-dependent manner, with the LTE50 group exhibiting a more pronounced reduction. This suppressive effect was particularly evident at a higher methacholine concentration.

### Effects of LTE on the inflammatory cell count of the BALF from the asthmatic mice

**Figure 4:**
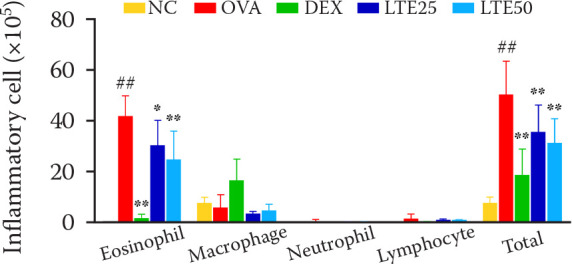
The effects of LTE on inflammatory cells in the BALF of OVA-inhaled animals The administration of LTE decreased the inflammatory cells in the BALF from the OVA inhaled animals. NC, PBS treatment only; OVA, OVA exposure animals with the oral administration of PBS; DEX, OVA exposure animals with the oral administration of dexamethasone; LTE25 and LTE50, OVA exposure animals with the oral administration (25 and 50 mg/kg, respectively) of LTE The values are displayed as the mean ± SD; *^##^P <* 0.01 versus NC; *P < 0.05, **P *<* 0.01 versus OVA BALF = bronchoalveolar lavage fluid; DEX = dexamethasone; LTE = *L. tanakae* ethanol extract; NC = normal control; OVA = ovalbumin; PBS = phosphate-buffered saline; SD = standard deviation

The total inflammatory cell count in the BALF was more elevated in the OVA group compared to the NC group, with a particularly marked increase in eosinophils (Figure 4). The DEX group showed a notable reduction in both total inflammatory cells and eosinophils compared with the OVA group. Likewise, the LTE treatment significantly decreased the number of inflammatory cells, including eosinophils.

### Effects of the LTE on the patho-physiological factors of the BALF and serum in asthmatic mice

**Figure 5:**
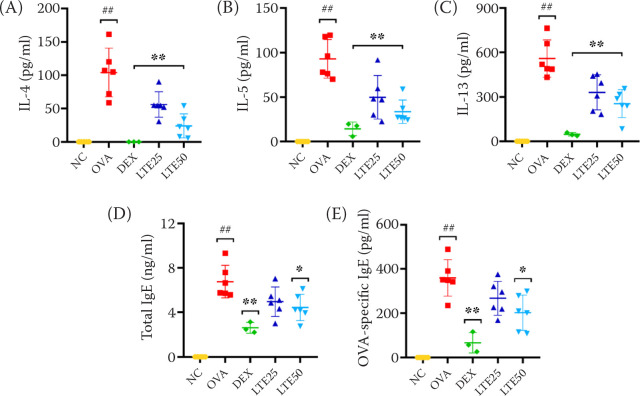
Effect of LTE on the pathophysiological factors of the BALF and serum from the OVA inhaled animals The administration of LTE decreased the levels of (A) IL-4, (B) IL-5, (C) IL-13, (D) Total IgE, and (E) OVA-specific IgE in the OVA inhaled animals. NC, PBS treatment only; OVA, OVA exposure animals with the oral administration of PBS; DEX, OVA exposure animals with the oral administration of dexamethasone; LTE25 and LTE50, OVA exposure animals with the oral administration (25 and 50 mg/kg, respectively) of LTE The values are displayed as the mean ± SD; *^##^P* *<* 0.01 versus NC; *P *<* 0.05, **P *<* 0.01 versus OVA BALF = bronchoalveolar lavage fluid; DEX = dexamethasone; IgE = immunoglobulin E; IL = interleukin; LTE = *L. tanakae* ethanol extract; NC = normal control; OVA = ovalbumin; PBS = phosphate-buffered saline; SD = standard deviation

The levels of IL-4, IL-5, and IL-13 were significantly higher in the OVA group than in the NC group (Figure 5).

These cytokines were markedly reduced in the DEX group and decreased in both LTE-treated groups, with a greater reduction observed in LTE50. Similarly, the total IgE and OVA-specific IgE were elevated in the OVA group, but significantly decreased in the DEX and LTE groups relative to OVA.

### Effects of the LTE on the pulmonary inflammation, mucus production and MMP-9 expression in asthmatic mice

**Figure 6:**
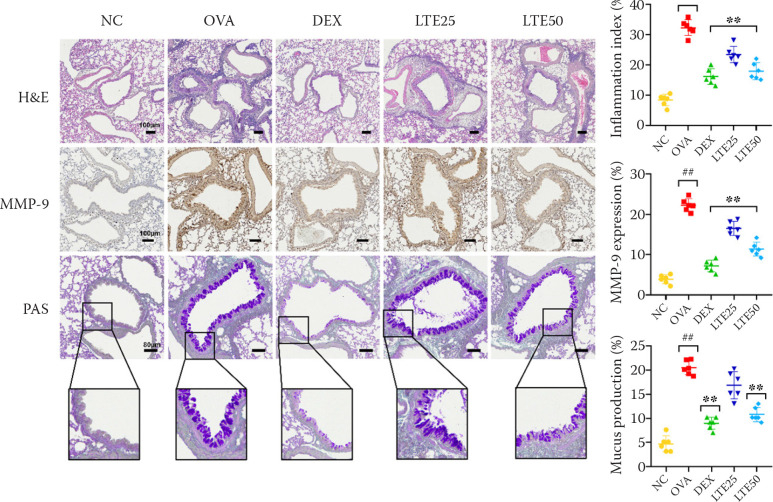
Effect of LTE on the pulmonary inflammation, mucus secretion, and MMP-9 in the lung tissue The administration of LTE alleviated the pulmonary inflammation and mucus secretion, with H&E (×80; Bar = 100 μm) and PAS (×150, ×400; Bar = 80 μm) staining confirming these effects individually, and it inhibited the expression of MMP-9 by IHC (×100; Bar = 100 μm). NC, PBS treatment only; OVA, OVA exposure animals with the oral administration of PBS; DEX, OVA exposure animals with the oral administration of dexamethasone; LTE25 and LTE50, OVA exposure animals with the oral administration (25 and 50 mg/kg, respectively) of LTE The values are displayed as the mean ± SD; *^##^P* *<* 0.01 versus NC; **P *<* 0.01 versus OVA DEX = dexamethasone; H&E = haematoxylin and eosin; IHC = immunohistochemistry; LTE = *L. tanakae* ethanol extract; MMP-9 = matrix metalloproteinase-9; NC = normal control; OVA = ovalbumin; PAS = periodic acid-Schiff; PBS = phosphate-buffered saline; SD = standard deviation

The histological analysis of lung tissue using H&E and PAS staining revealed significantly greater pulmonary inflammation in the OVA group than in the NC group (Figure 6). Both the DEX and LTE groups showed markedly reduced inflammation compared with the OVA group. Moreover, mucus hypersecretion was prominent in the OVA group, but substantially diminished in the DEX and LTE groups. The immunohistochemical analysis further demonstrated that the MMP-9 expression patterns corresponded with the extent of pulmonary inflammation and mucus secretion.

### Effects of the LTE on the MAPKs and MMP-9 of asthmatic mice

**Figure 7:**
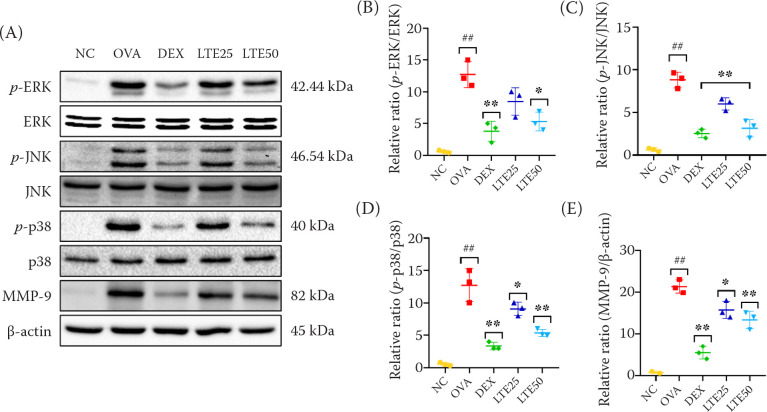
Effect of LTE on the inflammatory protein expression The administration of LTE downregulated (A) the expression of protein in the gel, and (B–E) the relative ratio of ERK, JNK, and p38 phosphorylation from each and expression of MMP-9. NC, PBS treatment only; OVA, OVA exposure animals with the oral administration of PBS; DEX, OVA exposure animals with the oral administration of dexamethasone; LTE25 and LTE50, OVA exposure animals with the oral administration (25 and 50 mg/kg, respectively) of LTE The values are displayed as the mean ± SD; *^##^P <* 0.01 versus NC; *P *<* 0.05, **P < 0.01 versus OVA DEX = dexamethasone; LTE = *L. tanakae* ethanol extract; MMP-9 = matrix metalloproteinase-9; NC = normal control; OVA = ovalbumin; PBS = phosphate-buffered saline; SD = standard deviation

The phosphorylation levels of ERK, JNK, and p38, key components of the MAPK signalling pathway, were pronounced increased in the OVA group compared with the NC group (Figure 7A). The administration of DEX or LTE resulted in a noticeable and measurable reduction in the phosphorylation of ERK, JNK and p38 (Figure 7B–D, respectively). In addition, the MMP-9 protein expression was markedly increased in the OVA groups compared with the NC group, while the LTE groups significantly and consistently decreased the MMP-9 expression levels notably compared with the OVA group (Figure 7E).

## DISCUSSION

Allergic asthma, a long-term respiratory disease, poses a serious threat to human health. Despite advances in treatment, there remains a continuing need to develop novel therapeutic agents, as currently available drugs for allergic asthma are limited in efficacy. LTE, a traditional remedy, has been used to treat various inflammatory conditions due to its diverse pharmacological properties. We appraised the therapeutic potential of LTE against an OVA-induced experimental allergic asthma model, and analysed its active components via HPLC. Quercitrin and afzelin were identified as the major constituents of LTE. In the allergic asthma model, LTE led to a significant decrease in the BALF inflammatory cell counts, which was accompanied by reductions in the AHR, proinflammatory cytokines, and OVA-specific IgE. The histological analysis demonstrated that LTE inhibited the accumulation of inflammatory cells and mucus secretion from the goblet cells in the lung tissue. Moreover, LTE effectively suppressed the MAPK phosphorylation in these mice.

Eosinophilic inflammation is a hallmark of asthma. Eosinophils contain numerous cytoplasmic granules enriched with biologically active molecules such as cytokines, growth factors, chemokines, tissue damaging protein, and reactive oxygen species (ROS) (Jackson et al. 2024; Wang and Liu 2024). These mediators are released either directly or indirectly contributing to the amplification of allergic inflammation in lung tissues (Ying et al. 1997). During the progression of allergic asthma, eosinophils infiltrate damaged tissues and are activated by cytokines such as IL-4, IL-5, and IL-13 (Figueiredo et al. 2023). IL-4 is pivotal in naive CD4^+^ T cells differentiating into Th2 cells, in the induction of IL-5 and IL-13 secretion, in promoting class switching in allergen-specific B cells, and in enhancing IgE release (Rosenberg et al. 2007). IL-5 facilitates eosinophil recruitment, maturation, activation and survival by modulating the intracellular signalling pathways including PI3K, JAK2, NF-κB and MAPKs (Hussain and Liu 2024). IL-13 contributes to asthma progression by upregulating the inducible nitric oxide synthase (iNOS) expression, driving excessive mucus production, and increasing AHR through bronchial smooth muscle contraction (Pak et al. 2022a). Activated eosinophils release these stimulatory factors, accelerating allergic asthma progression and leading to airway remodelling, enhanced AHR, and excessive mucus production (Hussain and Liu 2024). In the current study, the administration of LTE effectively reduced the number of inflammatory cells in the BALF of OVA-induced allergic asthmatic mice, along with a decline in IL-4, IL-5, and IL-13 production. Moreover, the LTE treatment markedly reduced inflammatory cell infiltration and mucus production in lung tissues. Taken together, these observations imply that LTE effectively suppresses the allergic responses in asthmatic animals.

Allergic asthma pathogenesis involves a complicated interplay of numerous signalling pathways (Shin et al. 2014). Of these signalling pathways, MAPKs are key players in regulating the inflammatory responses and innate immunity (Arthur and Ley 2013; Yang et al. 2021). During the progression of allergic asthma, various stimuli activate MAPKs through phosphorylation, which subsequently promote cell differentiation and stimulate the production of a broad array of inflammatory mediators, including cytokines, chemokines, growth factors, and matrix metalloproteinases (MMPs) (Alam and Gorska 2011; Khorasanizadeh et al. 2017; Saleem 2024). Among the MMPs, MMP-9 serves as a key indicator for assessing the severity of allergic asthma. As a proteolytic enzyme, MMP-9 degrades extracellular matrix proteins such as gelatin and collagen, which are essential for maintaining normal cellular architecture (Lee et al. 2024b). Under asthmatic conditions, MMP-9 is markedly upregulated in damaged lung tissue, disrupting alveolar structure and increasing production of inflammatory mediators, ultimately contributing to the loss of pulmonary function (Naik et al. 2017). Therefore, targeting MAPKs and MMPs is considered a critical therapeutic strategy in asthma management (Pelaia et al. 2005; Shin et al. 2012; Xu et al. 2023; Wang et al. 2024).

Through the present study, the phosphorylation of MAPKs – including ERK, JNK, and p38 – was effectively reduced by an oral LTE treatment in the allergic asthma model, with the concomitant suppression in the MMP-9 expression. These observations suggest a strong association between the anti-asthmatic effects of LTE and its ability to suppress MAPK signalling and MMP-9 expression. Prior research indicates that LTE exhibits a protective role in pathological respiratory conditions, including ASD-induced airway inflammation and CSC-induced COPD (Park et al. 2022; Lee et al. 2024a). Moreover, quercitrin and afzelin, two flavonoid compounds isolated from LTE, exhibit various pharmacological activities (Chen et al. 2022; Kciuk et al. 2024). Quercitrin is known to suppress immune activation and inflammatory responses by downregulating the MAPKs, T-bet, and GATA-3 (Li et al. 2016; Yu et al. 2022). Similarly, afzelin demonstrates anti-inflammatory properties by repressing MAPK signalling in particulate matter-exposed human keratinocytes and exhibits anti-asthmatic potential through the downregulation of GATA-3 in allergic asthma models (Zhou and Nie 2015; Kim et al. 2019). Furthermore, a recent study demonstrated that the combined administration of quercetin and kaempferol, the aglycones of these glycosides, effectively mitigates inflammation and oxidative stress (Mandal et al. 2025). Based on these findings, the potent therapeutic efficacy of LTE observed in this study may be attributable to the synergistic action of these active components. Future research investigating the individual and combined effects of these compounds would be beneficial for further elucidating the underlying mechanisms.

LTE effectively alleviates several pathological characteristics in an OVA-induced allergic asthma model including eosinophilic inflammation, cytokines and IgE secretion, airway hyperresponsiveness (AHR), and inflammatory cell infiltration in lung tissues. These effects were linked to the LTE modulating MAPK pathway. MMP-9 is a key effector in airway remodelling, specifically in the breakdown of type IV collagen and elastin, the disruption of epithelial integrity, and the release of TGF-β that stimulate fibroblasts, encourage smooth muscle hypertrophy, and increase extracellular matrix deposition. Crucially, the observed decrease in MMP-9 expression suggests that LTE may mitigate the airway remodelling caused by MMP-9. The significance of this study lies in its potential to position LTE as a useful therapeutic option for allergic asthma. Given the shortcomings of currently available drugs for allergic asthma, the potential efficacy of conventional remedies such as LTE offers encouraging insights for treatment. Collectively, our findings suggest that LTE is a promising option for the therapeutic management of allergic asthma.
